# Editorial: Big Data and Machine Learning in Cancer Genomics

**DOI:** 10.3389/fgene.2021.749584

**Published:** 2021-09-20

**Authors:** Lin Chen, Huimin Li, Longxiang Xie, Zhanjie Zuo, Liqing Tian, Changning Liu, Xiangqian Guo

**Affiliations:** ^1^Department of Preventive Medicine, Bioinformatics Center, Henan Provincial Engineering Center for Tumor Molecular Medicine, School of Basic Medical Sciences, Institute of Biomedical Informatics, Henan University, Kaifeng, China; ^2^Thoracic Cancer Treatment Center, Armed Police Beijing Corps Hospital, Beijing, China; ^3^Department of Computational Biology, St. Jude Children's Research Hospital, Memphis, TN, United States; ^4^CAS Key Laboratory of Topical Plant Resources and Sustainable Use, Xishuangbanna Tropical Botanical Garden, Chinese Academy of Sciences, Kunming, China

**Keywords:** OMICS data, profile, biomarker, therapeutic target, cancer

Cancer is one of the major causes threatening human health and life. With the rapid development of cancer genomics and bioinformatics analysis methods, a number of tumor biomarkers have been identified to facilitate the early detection, prognosis and treatment response prediction of tumors, and have successfully reduced the mortality of cancer patients (Wu and Qu, 2015). In recent decades, public profiling data sources, including the Gene Expression Omnibus (GEO) database and The Cancer Genome Atlas (TCGA) (Barrett et al., 2013) provide us the opportunities to explore the tumorigenesis and progression of cancers, and identify novel biomarkers for diagnosis, prognosis and treatment response. In this Research Topic of *Frontiers in Genetics* on Big Data and Machine Learning in Cancer Genomics, we have collected eight manuscripts that used single or multi-omics data to develop relative biomarkers for disease diagnosis, prognosis and treatment.

Cancer is a type of disease with high molecular heterogeneity that is a major cause of treatment failure. To elucidate the molecular heterogeneity of Endometrioid adenocarcinoma (EAC), Lei et al. used consensus clustering to analyze gene expression profiling data of EAC from TCGA and GEO and identified two different molecular subtypes (EAC I and EAC II), which were further verified in an independent EAC cohort. Moreover, three subtype specific diagnostic biomarkers including *MDM2* for EAC subtype I, *MSH2* and *MSH6* for EAC subtype II, were identified. This EAC subtyping would help to understand the mechanism of EAC tumorigenesis, and further facilitate the development of targeted therapies.

Prognostic biomarker can predict the outcome and help to guide the treatment of cancers. Benefiting from the recent advances of bioinformatics methods, Meng et al. analyzed the gene expression data of Clear Cell Renal Cell Carcinoma (ccRCC) cohort in TCGA and demonstrated that Caspase 4 (CASP4) (Shalini et al., 2015) could predict adverse overall survival (OS) of ccRCC patients and positively correlated with clinical stage and pathological grade. Functional enrichment analysis showed that the gene sets in the subgroup with higher *CASP4* expression were significantly enriched in the cell cycle and immune-related pathways. To deeply explore what components of the immune microenvironment were related to *CASP4*, they analyzed the proportion of tumor infiltrating immune cells (TICs) using CIBERSORT, and showed that activated CD4 memory T cells, follicular helper T cells, and regulatory T cells were positively correlated to *CASP4* expression. In addition, high expression of *CASP4* was found to be associated with drug resistance.

Although many single gene biomarkers have been reported, increasing studies demonstrated that multi-gene marker is more effective than single one even the cost of the multi-gene test is higher (Tao et al., 2020). Recurrence and metastasis are the main reasons of Prostate Cancer (PCa) patients' mortality. Thus, risk assessment methods are urgently needed to identify PCa patients at high risk of recurrence and metastasis (Lu et al., 2019). To solve this problem, Vittrant et al. used machine learning methods to develop a prediction model of a three-gene signature for PCa recurrence by in-depth analysis of transcriptome data. In addition, Zhang et al. analyzed the mRNA expression profiling and clinical histopathological data of breast cancers (BRCA) from TCGA, and identified four prognostic glycolysis genes, including *PGK1, SDHC, PFKL*, and *NUP43*. The high expression of the four genes, as an independent prognostic signature, could shorten the OS of BRCA patients.

Analysis of tumor genome, transcriptome and epigenome identified a number of tumor driver molecules (Argelaguet et al., 2018; Consortium ITP-CAoWG, 2020). So far, there are numerous bioinformatics tools available for gene expression profiling data analysis, however, the integrative analysis tools for multi-omics data are still limited. In this regard, Planell et al. designed a multi-omics conceptual framework (STATegra) by integrating three multi-omics methods (Component Analysis, Non-Parametric Combination, and an integrative exploratory analysis). STATegra not only saves time but also provides information that single mics cannot provide.

Recent reports showed that tumor microenvironment plays important regulatory roles in tumor progress and treatment resistance (Colli et al., 2017). More and more evidence of immune evade of TICs in the tumor microenvironment, have opened up the opportunities for developing therapies against the cross-talks between tumor cells and TICs, nowadays we call it immunotherapy, which has improved the prognosis of patients and provided the possibility of tumor remission in different types of cancers (Murciano-Goroff et al., 2020). To investigate the immune infiltration of lung squamous cell carcinomas (LSCC), Fu et al. collected the expression profiles of 502 LSCC and 47 adjacent normal tissues from TCGA, and identified seven immune-related prognostic genes (IRGs) including *GCCR, FGF8, CLEC4M, PTH, SLC10A2, NPPC*, and *FGF4*. In addition, they used CIBERSORT and TIMER to measure the infiltration levels of five immune cell types, including CD4 T cells, CD8 T cells, neutrophils, macrophages and dendritic cells, and showed a correlation of TICs with the patient's risk score.

Immune checkpoints regulate the intensity and extent of the immune response. During the development of tumors, the immune checkpoint has been evolved as one of the main causes of immune tolerance of cancers (de Miranda and Trajanoski, 2019). As a result, immune checkpoint inhibitor (ICI) has shown remarkable effects on the treatment of many cancer types, even though only a fraction of patients responded to ICI (Martins et al., 2019). To explore the incomplete response of ICI on bladder cancer patients, Yi et al. analyzed clinical and mutational data of 210 bladder cancer patients who had received immunotherapy, and demonstrated that bladder cancer patients with Ataxia Telangiectasia Mutated-mutant (ATM-MT) benefited from ICI treatment, and possessed longer OS, and may have increased sensitivity to 29 drugs.

Diagnostic markers are helpful to detect disease and guide the treatment in time. Preeclampsia (PE) is a major cause of maternal mortality. To identify the diagnostic biomarkers of PE, Wang et al. used machine learning methods and built a PE diagnostic signature, which could stratify PE into three subgroups with different clinical outcomes, may provide direction for individualized treatment of PE patients.

In summary, this Research Topic provides new bioinformatics tools and applications for omics data analysis and translational researches, paves the way for further development of tumor diagnostic, prognostic, treatment biomarkers, the tumor immune infiltrating estimation and immunotherapeutic treatment.

## Author Contributions

XG, CL, and LT conceived, designed, and supervised this project. LC, XG, CL, and LT wrote the manuscript. HL, LX, and ZZ revised the manuscript. All authors reviewed and approved the manuscript.

## Conflict of Interest

The authors declare that the research was conducted in the absence of any commercial or financial relationships that could be construed as a potential conflict of interest.

## Publisher's Note

All claims expressed in this article are solely those of the authors and do not necessarily represent those of their affiliated organizations, or those of the publisher, the editors and the reviewers. Any product that may be evaluated in this article, or claim that may be made by its manufacturer, is not guaranteed or endorsed by the publisher.
